# Supplementary material for: Efficacy of Modified Treat-and-Extend Regimen of Aflibercept for Macular Edema from Branch Retinal Vein Occlusion: 2-Year Prospective Study Outcomes
Source: J Clin Med. 2021 Jul 17;10(14):3162. doi: 10.3390/jcm10143162 (PMC8307685; doi:10.3390/jcm10143162)
Supplement: Supplementary file 1 [file jcm-10-03162-s001.zip › Supplement.pdf]

Efficacy of Modified Treat-and-Extend Regimen of Aflibercept for Macular Edema from Branch Retinal Vein Occlusion: 2-Year Prospective Study Outcomes

Yusuke Arai, MD[1]; Hidenori Takahashi, MD, PhD[1,2,3,*]; Satoru Inoda, MD [1]; Shinichi Sakamoto, MD[1]; Xue Tan, MD, PhD[2,3]; Yuji Inoue, MD, PhD[4]; Satoko Tominaga, MD[1]; Hidetoshi Kawashima, MD, PhD[1]; Yasuo Yanagi, MD, PhD[5]

[1] Department of Ophthalmology, Jichi Medical University, 3311-1 Yakushiji, Shimotsuke-shi, Tochigi 329-0498, Japan
[2] Japan Community Health Care Organization Tokyo Shinjuku Medical Center, 5-1 Tsukudocho, Shinjuku-ku, Tokyo 162-8543, Japan
[3] Department of Ophthalmology, Graduate School of Medicine, University of Tokyo, 7-3-1 Hongo, Bunkyo-ku, Tokyo 113-8655, Japan
[4] Department of Ophthalmology, Teikyo University, 2-11-1 Kaga, Itabashi-ku, Tokyo 173-8605, Japan
[5] The Ophthalmology & Visual Sciences Academic Clinical Program, Duke-NUS Medical School, National University of Singapore, 169857, Singapore

*Corresponding author: Hidenori Takahashi
Department of Ophthalmology, Jichi Medical University
3311-1 Yakushiji, Shimotsuke-shi, Tochigi 329-0431, Japan
Tel.: +81- 285-58-7382; Fax.: +81- 285-44-8365
E-mail: takahah-tky@umin.ac.jp
Tel.: +81- 285-58-7382; Fax: +81- 285-44-8365

E-mail: takahah-tky@umin.ac.jp

Supplementary Figure legends

Supplementary Figure S1-3 showed example of administration method of modified treat-and-extend regimen. Supplementary Figure S4 showed example of administration method of PRN and treat-and-extend regimen.

Supplementary Figure S1; The case of first retreatment criteria at month 3.

Supplementary Figure S2; The case of first TAE start criteria at month 4.

Supplementary Figure S3; The case of first retreatment criteria at month 10.

Supplementary Figure S4; (a) PRN regimen. (b) TAE regimen.
