# Supplementary material for: Efficacy of Modified Treat-and-Extend Regimen of Aflibercept for Macular Edema from Branch Retinal Vein Occlusion: 2-Year Prospective Study Outcomes
Source: J Clin Med. 2021 Jul 17;10(14):3162. doi: 10.3390/jcm10143162 (PMC8307685; doi:10.3390/jcm10143162)
Supplement: Supplementary file 1 [file jcm-10-03162-s001.zip › Supplymentary Figure.pdf]

Pattern 1: First retreatment criteria at month 3

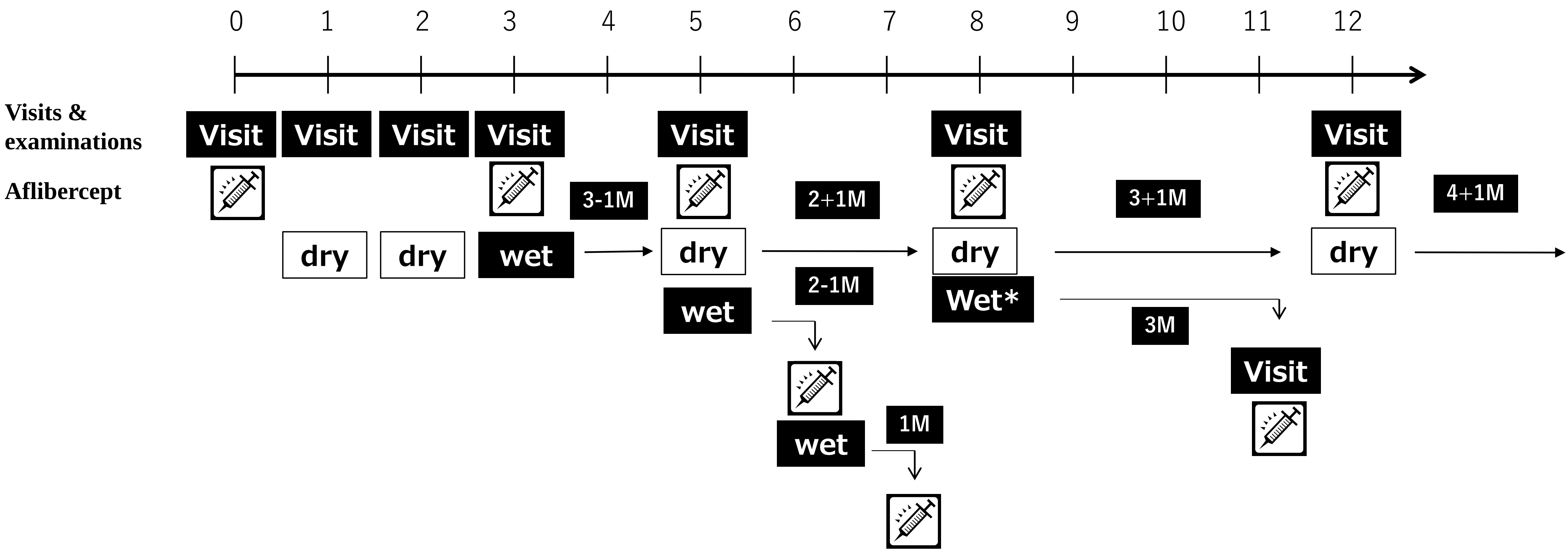

*the exudative change was the same ore less compared with the first recurrence

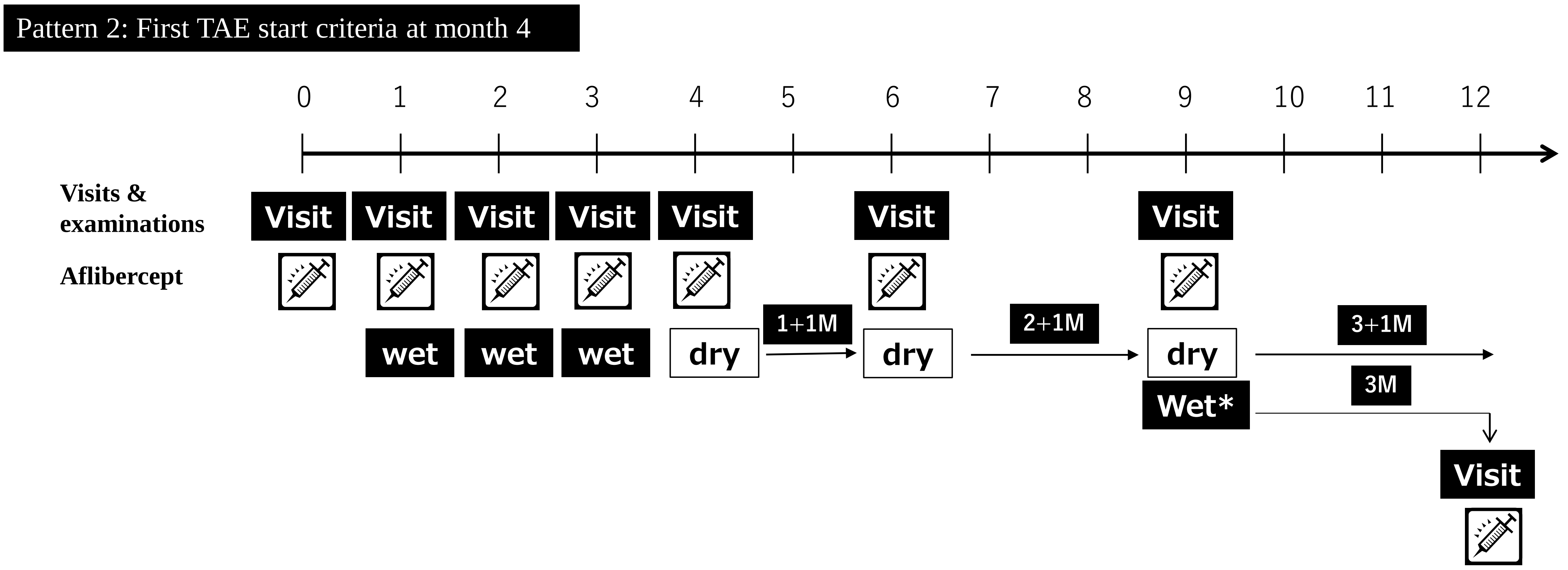

*the exudative change was the same ore less compared with the first recurrence

Pattern 3: The recurrence was occurred after the end of monthly examinations.

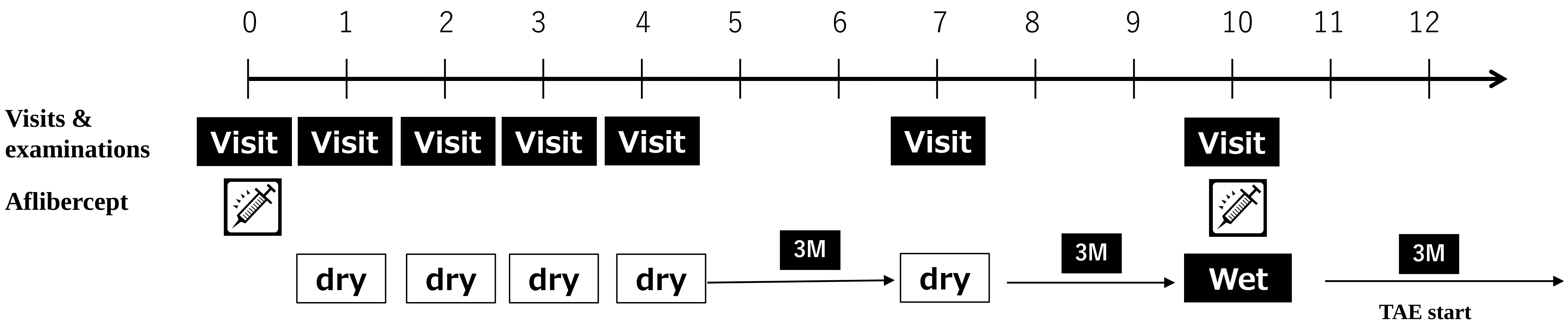

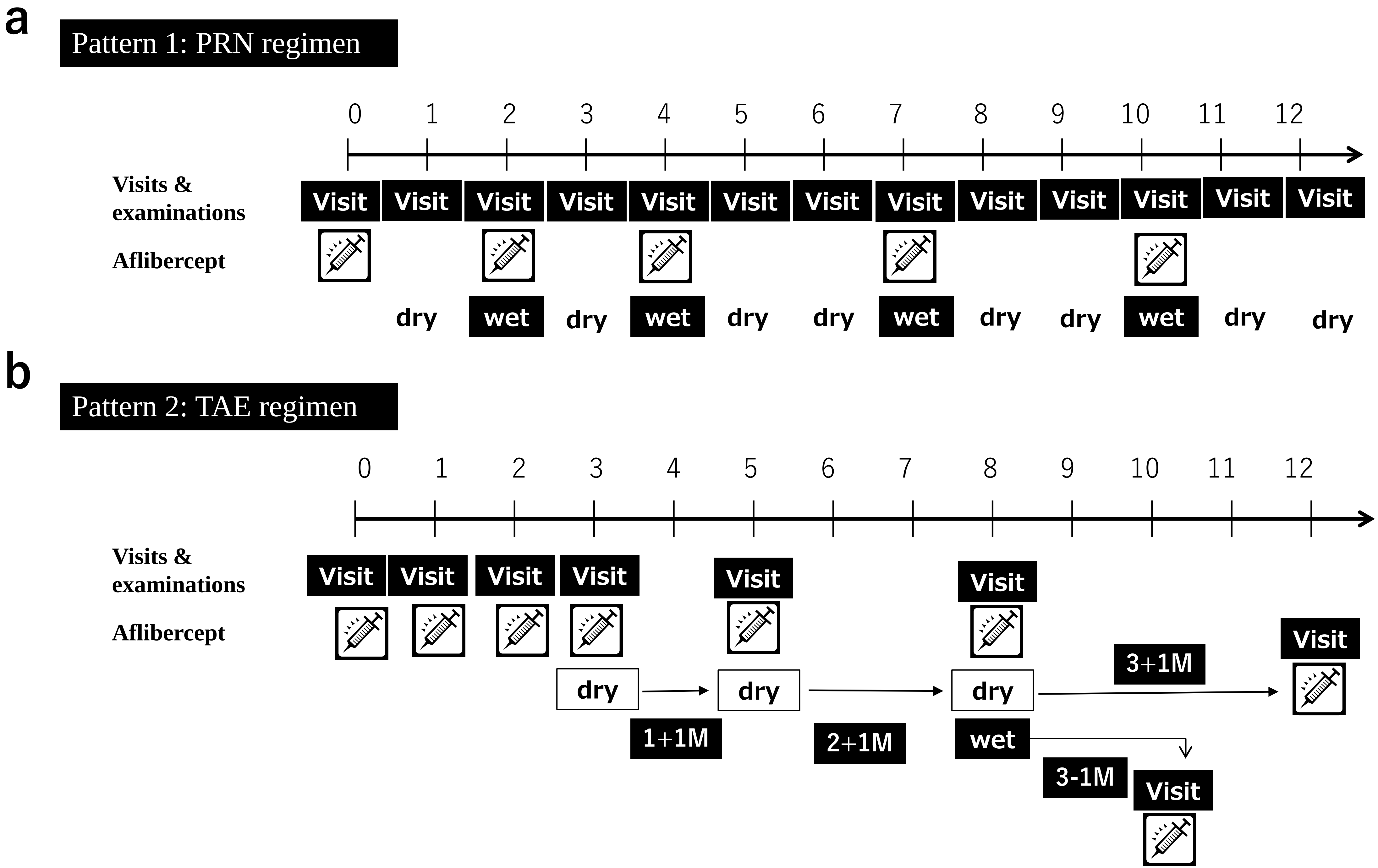
a
Pattern 1: PRN regimen
0 1 2 3 4 5 6 7 8 9 10 11 12
Visits & examinations
Visit Visit Visit Visit Visit Visit Visit Visit Visit Visit Visit Visit Visit
Aflibercept
dry wet dry wet dry dry wet dry dry wet dry dry
b
Pattern 2: TAE regimen
0 1 2 3 4 5 6 7 8 9 10 11 12
Visits & examinations
Visit Visit Visit Visit Visit Visit Visit
Aflibercept
dry
1+1M
dry
2+1M
dry
3+1M
wet
3-1M
Visit
Visit
